# Traumatic Spinal Cord Injury: A Contemporary Review of Classification, Evaluation, and Acute Management

**DOI:** 10.3390/jcm15166175

**Published:** 2026-08-10

**Authors:** John Carlos McDearman, John Attelah, Ali Nourbakhsh

**Affiliations:** 1 Jack Hughston Memorial Hospital, 4401 Riverchase Dr., Phenix City, AL 36867, USA; jmcdearman@jhmhospital.com; 2 Department of Medical Education, Morehouse School of Medicine, 720 Westview Dr., Alanta, GA 30310, USA; 3 OrthoAtlanta, 1240 Eagles Landing Pkwy, Stockbridge, GA 30281, USA

**Keywords:** humans, spinal cord injury, trauma, management, imaging, classification, methylprednisolone, surgery, literature review

## Abstract

Traumatic spinal cord injury (SCI) is a catastrophic condition associated with profound neurologic disability, psychosocial disruption, high long-term healthcare utilization, and reduced life expectancy. Globally, SCI remains a major cause of morbidity, most commonly resulting from traumatic mechanisms such as motor vehicle collisions and falls, with a bimodal age distribution affecting both younger adults and older individuals. This narrative review provides a comprehensive, clinically oriented overview of SCIs with emphasis on classification, initial evaluation, imaging, and contemporary management strategies. The review highlights the dual-phase pathophysiology of SCIs—primary mechanical insult followed by secondary injury cascades—underscoring the rationale for rapid stabilization and timely, injury-specific interventions. Initial care is framed around immobilization and trauma resuscitation principles, with cervical spine clearance guided by validated decision tools and early, appropriate imaging acquisition. We discuss adjunctive imaging considerations and address the ongoing debate regarding MRI use in obtunded patients with negative initial studies. Acute inpatient management priorities include prevention of secondary complications, hemodynamic optimization, respiratory support, and vigilant surveillance for autonomic dysfunction. Pharmacologic neuroprotection with high-dose methylprednisolone remains controversial; current guideline recommendations are reviewed. Finally, we outline operative decision-making and evidence supporting early decompression in selected patients, as well as common surgical approaches and their indications. This review consolidates foundational concepts and current standards to support practical, evidence-informed SCI evaluation and management.

## 1. Introduction

Damage to the spinal cord, whether caused by trauma or an underlying disease, is devastating and life-altering. Resulting neurological injuries have the potential to affect motor, sensory, and autonomic systems. Depending on a variety of factors, patients with spinal cord injuries (SCIs) may have resulting physical impairments that often necessitate prolonged dependence, lead to the development of social and psychological dysfunction, incur considerable cost, and are associated with significantly shorter life expectancies. In 2016, an estimated 282,000 people in the United States alone were living with SCIs, with an annual incidence of 17,000 new cases [1]. Global estimates vary widely, with reported prevalence ranging from 236 to 1298 per million people and annual incidence ranging from 9.2 to 246 per million, reflecting substantial geographic variation [2].

The economic burden of SCIs is substantial and disproportionately high compared to other pathologies [3]. Estimated lifetime financial burden per person with SCI in the U.S. ranges from $1.1 to $4.7 million depending on the age of onset, level of injury, and extent of deficits [1]. A cost analysis in veterans treated for SCIs also found cervical level injury, complete injury, and presence of pressure ulcers significant determinants of increased cost [4]. In 1995, the annual direct cost of SCIs in the U.S. was estimated at $7.7 billion, or roughly $15.8 billion today [5]. The global economic burden is more difficult to determine as there is significant heterogeneity in the literature and data are not available for all regions. A systematic review by Malekzadeh et al. found that healthcare costs are higher in developed countries, with the U.S. being the most costly, which further limits the generalizability of most estimates [3].

Trauma is far and away the leading cause of SCI, with motor vehicle accidents and falls being responsible for roughly 38% and 30%, respectively, of all SCIs in the U.S. in 2016. There exists a bimodal age distribution with peaks during young adulthood and in those greater than 65 years of age. Non-traumatic causes, such as medical conditions and iatrogenic complications, comprised less than ten percent [1,6]. Substantial variation exists in the etiology of injuries from demographic and temporal factors such as age, gender, and specific day of the week [6]. Global data is consistent with that collected in the U.S. [7].

This narrative review provides a broad overview of the classification, evaluation, and early management of traumatic spinal cord injury, from the field to the operating room. We discuss pertinent spinal cord injury classifications, injury patterns, principles of initial assessment and stabilization, imaging recommendations, and contemporary evidence guiding acute medical and surgical management. In addition, we briefly discuss the pathophysiology of primary and secondary spinal cord injury, indications for operative intervention, and commonly utilized surgical approaches. The goal of this review is to summarize current evidence and provide clinicians involved in the acute care of patients with traumatic spinal cord injury a practical framework for evaluation and management.

## 2. Methods

This manuscript was prepared as a narrative review of the contemporary literature on traumatic spinal cord injury. The literature was identified through searches of PubMed/MEDLINE and Google Scholar conducted between October 2024 and July 2026. To curate the list of articles for formal review, the authors identified landmark publications, clinical practice guidelines, and influential review articles addressing the major topics included in this manuscript. Additional relevant studies were identified through review of the references cited within these publications, allowing expansion of the literature to include foundational studies as well as more recent evidence. Priority was given to articles with direct clinical relevance to the classification, evaluation, acute management, and surgical treatment of acute traumatic spinal cord injury.

## 3. Spinal Cord Injuries: Classification

The vast collection of efferent and afferent tracts of the spinal cord, such as the corticospinal (pyramidal) tract that transmits signals for movement, extrapyramidal tracts that are pivotal in maintaining posture, balance, and motor tone, and the spinothalamic tract that carries those for pain and temperature, collectively comprise the predominant means by which information travels between the central and peripheral nervous systems. As various segments of the spinal cord are damaged throughout its length and cross-sectional anatomy, different sequelae of symptoms are seen. A robust understanding of the cord’s anatomy and tract decussations has led to the description of syndromes and injury patterns that effectively localize insults. In this section, we will briefly highlight several of these. Additionally, it is important to highlight that the pathophysiology of acute SCI can be attributed to both primary and secondary injury. The primary injury to the cord occurs immediately as a direct result of the injury. These injuries involve direct compression, impaction, distraction, or laceration of a distinct region of the cord by either bone, bone fragments, herniated intervertebral disk or disk contents, or foreign bodies with immediate evidence of neurologic injury.

On the other hand, secondary injury evolves over hours to weeks and consists of a complex cascade of biochemical and cellular events that occur as part of the innate response to the primary injury. Although these processes represent the body’s physiologic response to injury, their activation is ultimately detrimental to the surrounding spinal cord and contributes to progressive neurologic damage. Primary injury to the spinal cord disrupts the microvasculature, leading to hemorrhage, ischemia, and edema. In the acute phase, progressive edema impairs perfusion through adjacent intact microvascular networks, further compromising blood flow and exacerbating tissue injury. Cellular damage results in excessive glutamate release and excitotoxicity, leading to intracellular calcium accumulation, mitochondrial dysfunction, oxidative stress, and neuronal apoptosis. Axonal injury and neuronal apoptosis are followed by Wallerian degeneration, marking the transition into the subacute phase of secondary injury. These events trigger activation of astrocytes and resident microglia, as well as infiltration of peripheral inflammatory cells, resulting in the release of pro-inflammatory cytokines that further propagate the zone of injury. Reactive gliosis ensues, and eventual glial scar formation acts as a barrier to axonal regeneration and functional recovery. Although the primary injury is irreversible, timely medical and surgical management aims to mitigate these secondary processes and preserve viable neural tissue [8,9,10,11,12].

Generally, spinal cord injuries are separated into two categories: complete and incomplete injuries. Complete injuries are defined as injuries resulting in complete loss of both motor and sensory function below the level of the injury. Complete spinal cord injuries carry a poor prognosis with regard to clinically significant neurologic recovery. Therefore, when complete injuries are identified, focus often shifts toward managing and minimizing the sequelae associated with the injury. Incomplete injuries include those in which there are deficits in neurological function at or below the level of the injury, but with evidence of some degree of preserved function. These injuries are high-priority injuries and often necessitate timely and injury-specific management to prevent progression and maximize potential for neurologic recovery. The degree of recovery is highly variable and dependent on the level of injury, severity, and host factors affecting local biology [8,9,10,11].

Spinal cord injuries are further classified according to the American Spinal Injury Association (ASIA) Impairment Scale, or AIS, which is summarized in Table 1. The AIS grade is assigned using the International Standards for Neurological Classification of Spinal Cord Injury (ISNCSCI), which incorporates the level of injury, standardized motor and sensory examination, and assessment of sacral sparing [12,13,14]. The neurological level of injury is defined as the most caudal spinal segment with normal bilateral motor and sensory function. Sacral sparing distinguishes complete (AIS A) from incomplete spinal cord injury (AIS B-D). It is defined as some degree of retained motor and/or sensory function from the S4–S5 nerve roots. Complete cord injuries result in no sacral sparing. Therefore, if patients demonstrate any degree of retained perianal sensation (pinprick or light touch), retained sensation of deep anal pressure, or the ability to voluntarily contract the external anal sphincter, then their injury is considered incomplete (AIS B-D) [14].

Together, motor and sensory scores, the neurological level of injury, and sacral sparing are used to characterize the severity of neurologic impairment, facilitate communication, and guide subsequent principles of management. Importantly, the AIS classification is distinct from the anatomical incomplete spinal cord syndromes discussed below. Syndromes such as central cord syndrome, anterior cord syndrome, and Brown-Séquard syndrome describe characteristic patterns of neurologic dysfunction and injury localization rather than representing separate AIS classifications [12].

### 3.1. Complete Cord Injuries

A complete spinal cord injury, classified as AIS grade A, occurs when no cord function is observed below the level of injury. Motor, sensory, and other sympathetic and autonomic functions are lost. Loss of function at S4–S5 has been shown to be a reliable indicator of complete injuries [15]. These patients will also exhibit absence of reflexes, including the bulbocavernosus reflex, and male patients may present with priapism secondary to the loss of sympathetic input to the pelvic vasculature. These are, as the name implies, the most devastating of all SCIs and carry a poor prognosis.

The cervical spine is the most common site of traumatic injury, and roughly 17.3–34.0% of patients with cervical spinal cord injury present with complete cord injuries. The thoracic spine is the next most commonly injured level, and an estimated 16.2–73% of patients with thoracic spinal cord injuries present with complete injuries [16,17]. The incidence of complete injuries drastically increases at both levels in the setting of polytraumas [18]. As the spinal cord terminates between L1 and L2, low thoracic and lumbar spine injuries impact the conus medullaris and cauda equina, leading to muscle weakness, bladder, bowel, and sexual dysfunction [19,20]. In the setting of trauma, it is rare for injuries to the conus and cauda equina to occur separately, and they can be difficult to distinguish [21]. Although data are scarce, both cauda equina syndrome and conus medullaris syndrome are rare, impacting an estimated 3.4 and 1.5 in 1 million people, respectively [22].

Complete injuries carry a poor and highly variable prognosis, and initial trauma survey is paramount in establishing an accurate neurological baseline [23]. Meaningful recovery rates are defined as conversion from AIS grade A to AIS grade D, and have been reported in only 2.3% of patients with complete injury [24].

### 3.2. Incomplete Cord Injuries

Incomplete spinal cord injuries are classified as AIS grades B–D, and are characterized by preservation of some degree of neurologic function below the level of the injury. Loss of motor function is typically more pronounced than the loss of sensation. Due to the complex pathological and injurious cascade of events that follow acute spinal cord injury, it is imperative to identify and appropriately manage these injuries in a timely manner to prevent further injury and promote the possibility of recovery of function. These injuries are further classified according to the specific region of the spinal cord that is affected.

### 3.3. Central Cord Syndrome

Acute traumatic central cord syndrome (ATCCS) is the most common incomplete SCI pattern and has an annual incidence of 11 thousand cases in the United States. It represents an estimated 9.2% of all spinal cord injuries and 70% of all incomplete cord injuries [25]. ATCCS primarily affects males in a bimodal distribution, with over 90% of injuries occurring in persons over the age of 40. The first peak consists primarily of severe traumatic spinal cord injuries, while the second peak is presented by the classic hyperextension injury of an arthritic spine [26]. Patients present with motor dysfunction that is more pronounced in the upper extremities, particularly the hands. Bladder dysfunction is also commonly seen, although sacral sparing is present. Patients generally demonstrate varying degrees of touch, pain, and temperature sensory loss, as well. Damage to the anterior white commissure may present with a loss of pain and temperature sensation in a “cape-like” distribution over the shoulders and upper extremities [27].

The most common mechanism of injury is forced hyperextension of the neck following forward fall onto the chin. Spinal cord dysfunction following central cord injury has traditionally been thought to result from compression of vertebral disk/osteophytes anteriorly and anterior protrusion of the ligamentum flavum posteriorly, followed by edema and hemorrhage in the central cord at the site of compression. Return of neurological function has been shown to be closely associated with patient age. Patients under the age of 65 demonstrate the highest likelihood of regaining the ability to walk without assistive devices [28].

The pathophysiology of central cord syndrome has been the subject of much interest. It was hypothesized by Schneider et al., who originally described ATCCS in 1954, that the pattern of deficits seen is due to the organization of axons in the lateral corticospinal tract, where the axons that control muscles of the upper limbs and hands ascend more medially and are therefore disproportionately affected. Hematomyelia following injury was thought to facilitate injury to these central structures via vascular disruption and ensuing mechanical compression [29]. However, neuroanatomic tracer studies in primates have demonstrated that the fibers supplying the upper and lower limbs are diffusely dispersed in the corticospinal tract [30]. It is thought then that the relative density, rather than location, of fibers that control the upper limb contributes to the differential in weakness. Additionally, a post-mortem histological study in eleven patients with ATCCS revealed that the central gray matter remained intact without evidence of spinal cord hemorrhage, suggesting central hemorrhage is not a primary driver of the development of ATCCS [31]. Damage was seen diffusely through the axons of the white matter, primarily within the lateral columns, wherein the corticospinal tract ascends. This evidence brings into question our understanding of central cord syndrome and its supposed pathophysiology, highlighting the need for continued research as we continue to develop our understanding of this complex injury pattern [32].

### 3.4. Anterior Cord Syndrome

Anterior cord syndrome describes injury affecting the anterior two-thirds of the spinal cord. This area is a watershed region primarily supplied by the anterior spinal artery (ASA); therefore, anterior cord syndrome most often occurs in concert with injury to the ASA. Injury to the anterior cord is typically symmetric bilaterally (due to the vascular etiology), and therefore the location of axon decussation does not affect deficit pattern. Deficits typically include motor paralysis at and below the level of injury (typically more pronounced in the lower extremities as UMN lesion syndrome) and bilateral loss of pain and temperature sensation at and below the level of injury. Sensation to light touch and proprioceptive capability is characteristically preserved due to retained functionality of the dorsal column medial lemniscus. Autonomic dysfunction has also been observed, primarily in cases with injury to the thoracolumbar cord [33,34].

The ASA supplies the anterior two-thirds of the spinal cord, which includes the anterior horns and corticospinal tracts that house somatic motor neuron cell bodies and axons, respectively, the lateral horns from T1–L2 that contain sympathetic neuron cell bodies, and the afferent spinothalamic tracts that relay pain and temperature from the body [35]. Afferent sensations transmitted through the dorsal columns supplied by the posterior spinal arteries remain intact.

Anterior cord syndrome is rarely identified in isolation in the setting of acute traumatic SCI. Anterior cord syndrome is more commonly an iatrogenic complication of aortic aneurysm repair, cross-clamping the aorta, and hypotension during surgery. However, the etiology varies widely, as there are many medical conditions that can provoke spinal cord ischemia [35].

Despite being the most common form of spinal cord infarction, representing roughly 1.2% of all strokes [36], there is little epidemiological data on anterior cord syndrome—especially with specific focus on injuries occurring in the setting of acute trauma. Consequently, many published estimates are derived from patients with ischemic anterior cord syndrome secondary to anterior spinal artery infarction. One study estimates the incidence at 3.7 per 100,000 people in the United States [37] and reported mortality rates in the literature range from 9% to 23% [38,39].

### 3.5. Brown-Séquard Syndrome

First described in 1849 in a report of a sea captain who was stabbed in the neck, Brown-Séquard syndrome describes hemisection of the spinal cord and classically has been seen to result following ballistic injuries [40]. This pattern presents with ipsilateral loss of motor function below the injured spinal level, coinciding with injury to the lateral corticospinal tract. Within the same distribution, there is also loss of vibratory sensation and proprioception secondary to injury to the ipsilateral dorsal column medial lemniscus. Loss of pain and temperature sensation is also observed at the spinal level of injury on the ipsilateral side. However, injury also leads to loss of these sensations on the contralateral side beginning 1–2 levels distal to the spinal level of injury, whereas ipsilateral pain and temperature sensation is preserved along these same levels. This pattern of deficits is pathognomonic and is secondary to injury to the spinothalamic tracts, which decussate in the spinal cord two levels above the level of entry via the anterior white commissure. Ipsilateral deficits are seen due to injury to neurons that decussate above the spinal cord, while a mix of ipsilateral and/or contralateral deficits are seen in those that decussate in the cord [34,41].

Motor neurons decussate in the pyramids of the caudal medulla well before they descend the spinal cord within the corticospinal tracts. Therefore, axons responsible for ipsilateral motor function traverse the same side of the spinal cord, resulting in loss of motor function ipsilateral to the injury. At the spinal level of injury, ipsilateral flaccid paralysis is seen due to injury to the lower motor neurons. Below the spinal level of injury, ipsilateral spastic paresis is seen due to injury to the upper motor neurons.

The path of afferent sensory fibers is less straightforward. Axons within the dorsal column are divided into either the cuneate fasciculus (laterally) or gracile fasciculus (medially) and ascend ipsilaterally before decussating post-synaptically in the lower medulla. These axons carry advanced sensations such as fine touch, proprioception, two-point discrimination, and vibratory sensation. Hemisection of the cord therefore results in ipsilateral loss of these senses at and below the level of injury. Therefore, patients demonstrate an ipsilateral expression of injury similar to the corticospinal tracts.

In addition to the dorsal column, afferent sensory fibers can also be found in the anterolateral column within the spinothalamic tract. Fibers within this tract ascend one to two levels in Lissauer’s tract before decussating and ascending contralaterally in the ventrolateral spinal cord white matter. The spinothalamic tract is responsible for relaying sensory information regarding pain, temperature, and crude touch. Therefore, hemisection of the spinal cord and subsequent injury to the anterolateral column results in contralateral loss of these senses beginning one to two spinal levels below the lesion. However, ipsilateral loss of pain and temperature sensations may occur at the level of the injury; these fibers are damaged before crossing midline. This characteristic dissociation—ipsilateral motor weakness and loss of vibration and proprioception with contralateral loss of pain and temperature sensation—is the hallmark of Brown-Séquard syndrome [34,41].

Additionally, autonomic sympathetic fibers that innervate the eye constitute a three-neuron chain that leaves the hypothalamus before descending to the lateral horns of C8 through T2 before ascending to join the abducens and ophthalmic branch of the trigeminal nerve. Therefore, a hemisection of the spinal cord at the level T1 or above leads to ptosis, miosis, and facial anhidrosis, a condition known as Horner syndrome [41]. This pattern is not present in lesions below this level.

Roughly 11 thousand new cases of Brown-Séquard syndrome are reported each year in the United States [13] and it is estimated that they comprise 4% of traumatic spinal cord injuries in Norway [42]. Return of neurological function is similar, though not as robust, as that seen in central cord syndrome.

### 3.6. Level of Injury

The level of injury determines the extent of deficits due to anatomic differences throughout the spinal cord at each respective vertebral level. We will briefly discuss patterns of deficits based on level of injury, starting with the cervical spine.

The cervical spine is the most prone to injury due to its lack of anatomic protection and high degree of flexibility. High cervical spine injuries are defined as injuries between C1 and C4. Depending on the nature and severity of injury, patients may suffer from a wide range of deficits including quadriplegia, respiratory arrest secondary to loss of diaphragmatic innervation, and/or loss of bladder and bowel continence, possibly necessitating 24 h care. In comparison, low cervical spine injuries are defined as injuries occurring between C5 and C8. These injuries typically do not affect the motor innervation of the diaphragm and have variable impact on motor control of the upper extremities. Patients with injuries to the lower cervical spine may retain enough function to manage independently with the assistance of special equipment.

Roughly 35% of spinal cord injuries occur in the thoracic spine [43]. However, these injuries have not received quite the attention in the literature compared to those in the cervical spine. Trauma is the primary etiology of thoracic spine injuries. Injuries can be classified as high or low depending on their location in the thoracic spine. However, there is paucity of literature to meaningfully differentiate outcomes with regard to high or low thoracic SCI [44]. There is also reason to partition the thoracic spinal cord into an intermediate segment from T4–T7. This segment is a watershed region reliant on vascular anastomoses, which may deleteriously impact neurologic recovery [45]. High T-spine (T1–T5) injuries can impact the motor control of the upper chest, mid-back, and abdomen. Low T-spine (T6–T12) injuries impair the motor function of the trunk.

Representing about 15% of all spinal cord injuries, even less research has been conducted on traumatic injuries occurring in the lumbosacral spine [46]. Injury to levels from L1–L5 may result in loss of motor function to the legs and hips, and loss of bladder and bowel control, while injury to the nerve roots in the sacral spine may include some impairment in hip and lower extremity muscles with loss of bladder and bowel function.

## 4. Initial Care/Survey

### 4.1. Cervical Collar and Spinal Board

The initial response to patients with suspected spinal cord injury involves rapid immobilization of the spine, which is usually achieved through the use of a cervical collar and placement on a rigid spine board [47]. The cervical collars are crucial in immediate immobilization and protect patients with possible acute spinal cord injuries from secondary injuries that may be sustained from any movement of an unstable spinal column and vulnerable cord. Moreover, using cervical collars can be useful in preventing quadriplegia, which may occur following secondary injury to the cervical spinal cord. It is estimated that 25% of spinal cord injuries occur following the initial injurious event [48]. There is no defined duration of time that is appropriate for a patient to remain in the cervical collar and spine board—instead, the best indication to remove the collar and board is only after all spinal injury and pathology has been ruled out via appropriate imaging. Timely assessment and administration of appropriate treatment is of utmost importance in these patients, not only to minimize neurological damage, but also to prevent the development of pressure ulcers. Studies have shown that the incidence of pressure ulcers in patients placed in cervical collars ranges from 6% to 38% [49].

### 4.2. In Hospital

Upon arrival at the hospital, care of patients with potential spinal cord injuries begins with stabilization and thorough assessment. Appropriate management of the patient’s airway, breathing, and circulatory status takes immediate precedence. In awake and alert patients, the need for imaging can be determined by one of two criteria: the National Emergency X-Radiography Utilization Study (NEXUS), which is described in Table 2, or the Canadian C-spine Rule (CCR). Though both are commonly used, the CCR has been shown to be more sensitive for the detection of cervical spine pathology than the NEXUS criteria. However, the NEXUS criteria may be easier to implement in the emergency setting [50]. The NEXUS criteria demonstrate a sensitivity of 99.6% for the detection of cervical spinal column injuries in awake and oriented patients under the age of 60. Of note, the sensitivity of this criterion drops in the elderly. However, by simply adding injury to the face or head and deviations from baseline mental status as indicators for imaging, the sensitivity and negative predictive value increase to 100% in patients >65 years of age [51]. In those who fail to satisfy the NEXUS criteria, CT imaging is the first-line modality for evaluating cervical spine injuries in awake and symptomatic patients [52]. If providers garner a high clinical suspicion for spinal injury, neurology or spine services should be consulted regardless of the results from imaging studies. Patients may not be removed from cervical collars and the spine board until imaging studies have been confirmed as negative by a radiologist [8,50].

In patients that do not qualify for use of the aforementioned criteria (i.e., those with GCS < 15, including obtunded patients), illicit clinical suspicion of spinal cord injury, or fail criteria for conservative management, imaging should include a trauma series CT. This series includes a CT brain without contrast, CT cervical spine without contrast, and CT chest, abdomen, and pelvis [8]. Although plain radiographs are often obtained during the initial trauma evaluation, they provide little additional value beyond the early identification of gross fracture-dislocations when high-quality CT is available. The indications for obtaining additional modalities such as dynamic imaging and MRI in obtunded patients have not been fully elucidated. Dynamic imaging on the obtunded patient carries significant risk of secondary injury, and the literature has demonstrated conflicting evidence on the use of MRI to further assess for SCI in the setting of a negative initial high-quality trauma series CT [48].

MRI is complementary to CT and should be considered in stable patients with clinical signs or symptoms of neurologic injury following consultation with the on-call spine surgeon. MR imaging provides additional information which, in the appropriate patients, may influence management, as it allows for enhanced evaluation of soft tissue and the internal structure of the spinal cord. Indications include unexplained neurologic deficits in the setting of negative CT imaging and in those with suspected spinal column ligamentous injury. MRI is also indicated for preoperative planning in stable patients prior to surgical intervention, as it allows for characterization of spinal cord edema/hemorrhage, epidural hematomas, and traumatic disk herniations—all of which contribute prognostic value and may affect surgical decision-making [8,51,53].

The modified Denver screening criteria are an additional set of screening guidelines designed to identify those at risk for vascular injury and for whom further imaging is indicated, as illustrated in Table 3. CT angiography (CTA) has been shown to have high sensitivity and specificity for detecting vascular injury in these patients and is the gold standard imaging modality at this time.

Patients with confirmed spinal cord injuries require consistent monitoring for the development of potentially fatal sequelae. Common complications of SCI include hypotension secondary to disruption of the autonomic nervous system and pulmonary complications secondary to injury to either the phrenic nerve or the C3–C5 nerve roots [8,54]. Hemodynamic optimization is a heavily researched and debated aspect of acute SCI management. Current clinical practice guidelines recommend avoiding hypotension and maintaining the mean arterial pressure (MAP) between 75 and 80 mmHg and 90–95 mmHg during the first 3–7 days following the injury to optimize spinal cord perfusion. In those presenting with a MAP above 95 mmHg in the absence of vasopressor augmentation, no intervention is necessary. Classically, dopamine or norepinephrine is used to maintain the MAP. Studies have demonstrated increased adverse events with use of dopamine in comparison to norepinephrine in the elderly; however, the choice of vasopressor or ionotrope should be tailored to the patient based on hemodynamic status and comorbidities [54,55]. In patients with injuries above T6, medications that may cause reflex bradycardia are contraindicated [8]. For those with signs of respiratory compromise, orotracheal intubation is safe and effective when performed with proper inline cervical stabilization [48]. Due to the need for attentive cardiopulmonary observation and timely response to changes in status, it is often recommended that patients spend a minimum of 5 days in the ICU during initial stabilization to minimize the risk of morbidity from secondary complications.

The use of steroids in patients with acute traumatic spinal cord injuries is a heavily debated topic. The National Acute Spinal Cord Injury Studies (NASCIS I, II, and III) were performed to study the optimal timing and dosing regimens of methylprednisolone associated with improved outcomes [56,57,58]. These studies demonstrated that the potential benefits from the administration of methylprednisolone are variable and mild. If administered within 8 h of injury, there were mild improvements in motor function in patients receiving methylprednisolone; however, these differences were not statistically significant at 1-year follow-up [58]. Additionally, multiple studies have shown that the administration of steroids following SCI may be associated with increased mortality [59,60]. Administration may place patients at higher risk of infection, respiratory complications, GI hemorrhage, and death [8,59,60]. The current AOSpine guidelines suggest administration of 24 h of high-dose methylprednisolone in adult patients who present within 8 h of acute SCI, but recommend against its administration in all other populations [61]. Accordingly, steroid administration in acute SCI is institution-dependent and, where utilized, is often limited to carefully selected patients presenting within a narrow therapeutic window.

### 4.3. Closed Reduction: Cervical Spine

In patients presenting with acute fracture-dislocations of the cervical spine, closed reduction may be attempted following appropriate and timely imaging and diagnosis. Closed reduction appears to be most effective when performed within 24 h of initial injury, although reduction for those who fall outside this window may still be feasible [62,63]. There are several methods for performing reduction, with the most commonly used being Gardner–Wells tongs and the Halo ring. There is debate regarding the use of anesthesia during reduction. Lee et al. (1994) published a cohort study consisting of 200 patients undergoing manipulation under anesthesia (MUA) or progressive, controlled craniocervical traction, and concluded that progressive, controlled traction had similar outcomes with a more favorable safety profile than those undergoing MUA [64]. Prior to reduction, a thorough neurologic examination should be documented and correlated with imaging findings to confirm the injury pattern and identify appropriate candidates for attempted closed reduction. Reduction should be performed by a multidisciplinary team with continuous neurologic and hemodynamic monitoring. Immediate operative stabilization should be available if closed reduction is unsuccessful or if neurological status deteriorates during the procedure [65,66].

The necessity for pre-reduction MRI is also debated. Pre-reduction MRI allows for assessment of disk material, and it is believed that closed reduction in the setting of significant disk herniation could lead to further impingement and neurological deterioration. However, acquiring pre-reduction MRI requires movement and risk of manipulation of an unstable spine and also prolongs time to reduction [48,65].

## 5. Surgical Treatment Options

### 5.1. Indications

Prompt surgical treatment is indicated in patients at risk for secondary spinal cord injuries, which may occur due to a robust inflammatory response following the initial injury. Timely diagnosis and implementation of appropriate management can mitigate the magnitude of the secondary injury and maximize the potential for neurologic recovery. Spinal cord compression and neurological deficits are the major deciding factors for surgery. The most appropriate surgical approach, which may include anterior, posterior, or both, is dependent on many factors, including level of injury, nature of instability (if present), and the anatomic location of inciting pathology. The goals of operative management in acute traumatic spinal cord injury are multifactorial and should be individualized based on the injury pattern and neurological status. Although these procedures are frequently performed in combination, decompression, reduction, stabilization, and fusion represent distinct surgical objectives. Depending on the injury pattern, one or more of these objectives may be achieved during a single operative procedure.

Surgical decompression is indicated within the first 24 h in patients who demonstrate progressive neurological deterioration, patients with evidence of ventral disk herniation risking vascular compromise, and those with a dislocation-type injury to the spinal column [67]. Early decompression may also be associated with improved neurologic outcomes in patients with complete cervical acute traumatic SCI [68]. However, any patient who may benefit from acute decompression and/or stabilization may be a candidate for surgical management [8,67].

The timing of surgical management has been a heavily researched topic in recent years. Badhiwala et al. (2018) published a meta-analysis examining the effect of surgical timing on patient outcomes at 1 year post-operatively in patients with acute traumatic spinal cord injury (n = 1548). They found statistically significant improvements in total motor scores, light touch scores, pin prick scores, and ASIA impairment scale grades at 1 year in patients receiving surgical decompression in <24 h. There was also a significant linear relationship between outcomes and timing of surgery in the 24–36 h window [69]. Dvorak et al. (2015) published a longitudinal cohort study that included 888 patients and found no significant difference in outcomes in those undergoing early (<24 h) versus late (>24 h) surgical intervention. However, when analyzed according to AIS subgroup, those patients with AIS B, C, and D injuries demonstrated statistically significant improvements in motor scores (approximately 6 points) when undergoing early surgical intervention. Granted, the clinical relevance of this finding is uncertain [70].

Unfortunately, it does not appear that early surgical intervention is a universally applicable determination. In patients with incomplete cervical SCI without bony injury and preexisting canal stenosis, treatment with early surgical decompression demonstrates quicker neurological recovery, but outcomes appear to be similar at 1 year [71]. In patients at high risk for surgical morbidity and lack of obvious indication, it may be appropriate to delay surgery to further assess the progression of neurological status and need for operative management. In summation, the benefits of early decompression should be interpreted within the context of the individual patient’s injury pattern, neurologic status, radiographic findings, and overall surgical risk.

### 5.2. Anterior Cervical Decompression and Fusion

The anterior approach to the cervical spine allows for direct neural decompression and, in select patients, facilitates reduction, stabilization, and fusion through a single approach. Indications for this approach include spinal cord compression from herniated disks or anterior bone fragments (usually secondary to burst fractures) [72,73]. This technique may also be preferred in patients with contraindications to being manipulated into prone positioning. Additionally, in patients with cervical spine injury, anterior decompression requires less muscle dissection and has also been associated with decreased operative times and blood loss when compared to the posterior approach [72,74,75,76]. Ren et al. (2020) also showed that the anterior approach provides improved cervical alignment at 2-year follow-up [76]. Though the anterior approach is primarily utilized for subaxial cervical spine pathology, recent data have suggested that an anterior approach may have a role in the treatment of injuries in the thoracolumbar region [75]. Neurological recovery benefits were reported by Bohlmann et al. (1992) in conus medullaris and cauda equina decompression [10].

### 5.3. Posterior Fusion

The posterior approach is the utilitarian approach to the spine and is regularly used in the treatment of cervical, thoracic, and lumbar spine pathology. In the cervical spine, utilization of the posterior approach is considered necessary in cases of unreduced facet dislocation and locking, posterior ligamentous disruption, or following failed stabilization via the anterior approach [72,73,74,76]. This approach has been shown to provide better overall biomechanical stabilization, but is associated with increased risk for wound infection when compared to the anterior approach [77,78,79,80].

In the setting of thoracic decompression, the posterior approach is favored largely due to surgeon training, comparative simplicity of dissection, and improved visibility. It is important to correct the dislocated vertebrae before considering fusion. However, large-scale studies have not been conducted to definitively assess the superiority of the posterior approach with respect to patient outcomes [75].

## 6. Conclusions

Spinal cord injury remains one of the most devastating conditions encountered in orthopedic and trauma care, with profound neurological, functional, psychosocial, and economic consequences. As outlined in this review, SCI encompasses a heterogeneous group of injuries that vary widely by mechanism, level, severity, and complexity, each carrying distinct prognostic and management implications. A clear understanding of spinal cord anatomy and injury classification is essential for accurate diagnosis, localization, and early decision-making.

Early recognition and prompt implementation of evidence-based management remain important modifiable determinants of neurologic outcome following acute traumatic spinal cord injury. Accurate classification, adherence to validated clearance and imaging protocols, prevention of secondary injury, and meticulous cardiopulmonary management form the foundation of acute care. Clinicians involved in the initial evaluation of these patients should be familiar with the NEXUS, Canadian C-Spine Rule, and modified Denver criteria in order to provide safe and timely care for these patients.

Surgical management continues to evolve, with increasing evidence supporting early decompression and stabilization in appropriately selected patients, particularly those with ongoing neurological compromise or mechanical instability. However, optimal timing, approach, and patient selection remain nuanced and must be individualized based on injury pattern, comorbidities, and overall clinical context.

Despite substantial advances in acute management, neurological recovery following traumatic SCI remains highly variable. Continued investigation into injury pathophysiology, neuroprotective strategies, and optimization of surgical management will further refine evidence-based care and improve outcomes for this complex patient population.

## Figures and Tables

**Table 1:** American Spinal Injury Association impairment classification.

Clinical Presentation	Grade
No preservation of function below the level of the injury, without sacral sparing (S4–S5).	A
Incomplete: Sensory function preserved, but with complete loss of motor function below the level of the injury. Sacral sparing is present.	B
Incomplete: Motor function is preserved below the neurological level, with more than half of the key muscle groups below the neurological level demonstrating a muscle grade <3.	C
Incomplete: Motor function is preserved below the neurological level, and at least half of key muscles below the neurological level demonstrate a muscle grade of ≥3.	D
Incomplete: Motor and sensory function are normal.	E

**Table 2:** The NEXUS criteria used to determine the need for imaging.

NEXUS Criteria ^1^
Criteria for clinical clearance of cervical immobilization device in an awake and asymptomatic patient
No midline cervical tenderness
No focal neurological deficit
Normal alertness and mentation
No intoxicants or confounders
No distracting injury

^1^ If the patient meets all 5 of the above criteria, the C-collar can be removed, and no additional Cervical spine imaging is necessary.

**Table 3:** The modified Denver screening criteria used for identifying those at increased risk of vascular injury and for whom further imaging is indicated.

Modified Denver Criteria ^2^
Criteria identifying patients that require screening for blunt cerebrovascular injury
Lateralizing neurologic exam unexplained by CT head
Infarct on CT head
Cervical hematoma
Epistaxis (massive)
Anisocoria/Horner’s Syndrome
GCS < 8 unexplained by head CT
Cervical spine fracture
Basilar skull fracture
Severe facial fracture
Supraclavicular seat belt sign
Cervical bruit/thrill

^2^ If patient meets any of the above criteria, CTA of the neck is indicated to evaluate for possible blunt cerebrovascular injury.

## Data Availability

No new data were created or analyzed in this study. Data sharing is not applicable to this article.

## Abbreviations

The following abbreviations are used in this manuscript:

SCI	Spinal Cord Injury
MRI	Magnetic Resonance Imaging
U.S.	United States
ASIA	American Spinal Injury Association
ATCCS	Acute Traumatic Central Cord Syndrome
ASA	Anterior Spinal Artery
UMN	Upper Motor Neuron
NEXUS	National Emergency X-Radiography Utilization Study
CCR	Canadian C-Spine Rule
GCS	Glasgow Coma Scale
CT	Computed Tomography
CTA	Computed Tomography Angiography
MAP	Mean Arterial Pressure
ICU	Intensive Care Unit
NASCIS	National Acute Spinal Cord Injury Studies
GI	Gastrointestinal
MUA	Manipulation Under Anesthesia
